# Synthesis of strong magnetic response ZIF-67 for rapid adsorption of Cu^2+^

**DOI:** 10.3389/fchem.2023.1135193

**Published:** 2023-03-16

**Authors:** Yuanhang Lei, Haibo Yang, Jiangqin Xie, Qi Chen, Wenxuan Quan, Anping Wang

**Affiliations:** ^1^ School of Materials and Architectural Engineering, Guizhou Normal University, Guiyang, Guizhou, China; ^2^ Key Laboratory for Information System of Mountainous Area and Protection of Ecological Environment of Guizhou Province, Guizhou Normal University, Guiyang, Guizhou, China

**Keywords:** magnetic, ZIF-67, rapid adsorption, Cu^2+^, MOFs adsorbent

## Abstract

With the acceleration of industrialization and urbanization, global water resources have been polluted. Among the water pollutants, heavy metals have caused great harm to the environment and organisms. When the concentration of Cu^2+^ in water exceeds the standard, the intake of the human body will mainly damage the nervous system. We use MOF materials with high chemical stability, specific surface area, adsorption, and other unique properties to adsorb Cu^2+^. MOF-67 was prepared with various solvents, and a stronger magnetic response MOF-67 with the largest surface area and best crystal form were selected. It quickly adsorbs low-concentration Cu^2+^ in water to purify water quality. At the same time, it can be recovered promptly through an external magnetic field to avoid secondary pollution, which conforms to the concept of green environmental protection. When the initial concentration of Cu^2+^ is 50 mg/L for 30 min, the adsorption rate reaches 93.4%. The magnetic adsorbent can be reused three times.

## 1 Introduction

In recent years, with the rapid development of science and technology, industrial growth has also accelerated, but industrial development has also left a heavy burden on the environment (He et al., 2016; Al-Qahtani et al., 2021; Jiang et al., 2021). The water quality of rivers and lakes in some areas is worsening, and heavy metals are detected to exceed the standard. The excessive heavy metals in water, especially Cu^2+^, will significantly impact the environment and endanger human health (Li et al., 2022). Therefore, developing an adsorbent that can quickly adsorb Cu^2+^ and is easy to recover is essential.

The methods for repairing heavy metal-polluted water mainly include chemical precipitation, redox, electrochemical, adsorption, membrane separation, and flocculation (Ulrich et al., 2015; Fox et al., 2016). Adsorption is a simple and low-cost treatment technology widely used in water treatment. Metal-organic framework materials (MOFs) are crystalline porous materials with periodic network structures formed by the self-assembly of transition metal ions and organic ligands. Because of its low density, high porosity, high specific surface area, and modifiability, it has essential applications in heavy metal adsorption. In previous reports, MOFs such as ZIF-8, ZIF-67, and UIO-66 are used for the adsorption of Cu^2+^, mainly because these materials are easy to synthesize and can exist stably in water (Goswami et al., 2020; Mao et al., 2021; Wang et al., 2022). Huang et al. synthesized the porous adsorbent of ZIF-67 and further studied its performance in removing Cu^2+^ from wastewater (Huang et al., 2018). The results show that the saturated adsorption capacity is much higher than other adsorption materials, and it shows that ZIF-67 is an excellent candidate for removing heavy metal ions from wastewater.

However, it is regrettable that the powder state of MOF is very unfavorable for its recovery. A small amount of wastewater can be recycled by filtration, but if it is large, it will consume a lot of workforce and material resources. Scientists have made various attempts, among which encapsulating magnetic nano ions with MOF materials is a good choice (Angamuthu et al., 2017; Aghayi-Anaraki and Safarifard., 2020; Song. et al., 2022). We designed to encapsulate Fe_3_O_4_ in ZIF-67 to form Fe_3_O_4_@ZIF-67. with solid magnetism so that it can be easily separated by an external magnetic field, which is undoubtedly gratifying.

In this paper, we first discussed the preparation of ZIF-67 in different solvents. Finally, we used ZIF-67 with methanol as the solvent with the best morphology and physical and chemical properties to encapsulate Fe_3_O_4_. Finally, Fe_3_O_4_@ZIF-67 material with excellent magnetic properties is used in the adsorption experiment of low-concentration Cu^2+^.

## 2 Materials and methods

### 2.1 Materials

2-Methylimidazole (AR, ≥98%), Sodium diethyldithiocarbamate (AR, ≥98%)**,** Cobalt nitrate hexahydrate (Co(NO_3_)_2_·6H_2_O, AR, ≥99.7%), Copper nitrate trihydrate (Cu(NO_3_)_2_·3H_2_O, AR, ≥99.7%), Sodium 3-diethyldithiocarbamate trihydrate (DDTC), Iron chloride hexahydrate (FeCl_3_·6H_2_O, 99%), sulfate heptahydrate (FeSO_4_·7H_2_O, ≥98.0%), ammonia solution (AR, 25.0%–28.0%) were obtained from Shanghai Macklin Biochemical Co., Ltd. Absolute methanol (AR, ≥99.5%), Absolute ethanol (AR, ≥99.5%) were purchased from Tianjin Zhiyuan Chemical Reagent Co., Ltd.

### 2.2 Adsorbents preparation

#### 2.2.1 Preparation of ZIF-67

48 mmol of 2-methylimidazole and 12 mmol of cobalt nitrate hexahydrate are generally dissolved in 150 mL of methanol, respectively. Then, mix the two solutions, stir them at 25°C for 30 min, and let them stand for 24 h to obtain a blue-purple turbid solution. Finally, ZIF-67 (methanol) was obtained after centrifugation, washing, and drying (Li et al., 2016). When absolute ethanol and distilled water are used as solvents, the experimental method and dosage are the same as that of absolute methanol.

Preparation of Fe_3_O_4_@ZIF-67: Fe_3_O_4_ was prepared by the coprecipitation method concerning the literature (Wang et al., 2018). The above form is used to prepare Fe_3_O_4_@ZIF-67. The only difference is that 2.0 g of Fe_3_O_4_ is added to the 2-methylimidazole solution.

### 2.3 Adsorbents characterization

The XRD, FT-IR, N_2_ adsorption and desorption, SEM, TEM, and VSM were used to characterize the adsorbents. XRD (Cu K α radiation *λ* = 0.154,056 nm) reflects the diffraction peak of the adsorbent at 10–80°, and FT-IR (360 Nicolet) gives the absorption spectrum band of the adsorbent at 500–4,000 cm^-1^. However, N_2_ adsorption-desorption (Micromeritics Co., Ltd., ASAP 2460) provides information about specific surface area, pore diameter, and pore volume from adsorbents. The mass loss at 25–800°C in argon atmosphere (Mettler TGA/DSC1) was measured by thermogravimetric analysis (TGA). The heating rate is 10°C/min. SEM and TEM directly reflected the morphology of the adsorbent. The VSM provides the magnetic properties of the adsorbents.

### 2.4 Adsorption experiments

Put a specific volume of Cu^2+^ solution into a 250 mL conical flask, and then add Fe_3_O_4_@ZIF-67. Then, put it into a constant temperature oscillator to oscillate for some time, take out the magnetic separation adsorbent, measure the concentration of Cu^2+^ solution in the solution, and calculate the adsorption rate.

### 2.5 Determination of Cu^2+^ concentration in water

The concentration of a copper ion in water was determined according to the method of Water Quality Determination of Sodium Copper Diethyldithiocarbamate Spectrophotometry (HJ 485–2009, China) (Liu et al., 2018). Measure each sample three times in parallel and finally get the average value. In an alkaline ammonia solution, copper ion reacts with copper reagent (sodium diethyldithiocarbamate, abbreviated as DDTC-Na) to form a yellow-brown colloidal complex. When there is a certain amount of copper ion in the water, it can be determined by an ultraviolet visible spectrophotometer.

## 3 Results and discussion

### 3.1 Characterization of ZIF-67

As solvents, the MOF-67 was synthesized using methanol, ethanol, and distilled water. To distinguish, MOF-67 was labeled as MOF-67 (methanol), MOF-67 (ethanol), and MOF-67 (H_2_O), respectively.

**FIGURE 1:**
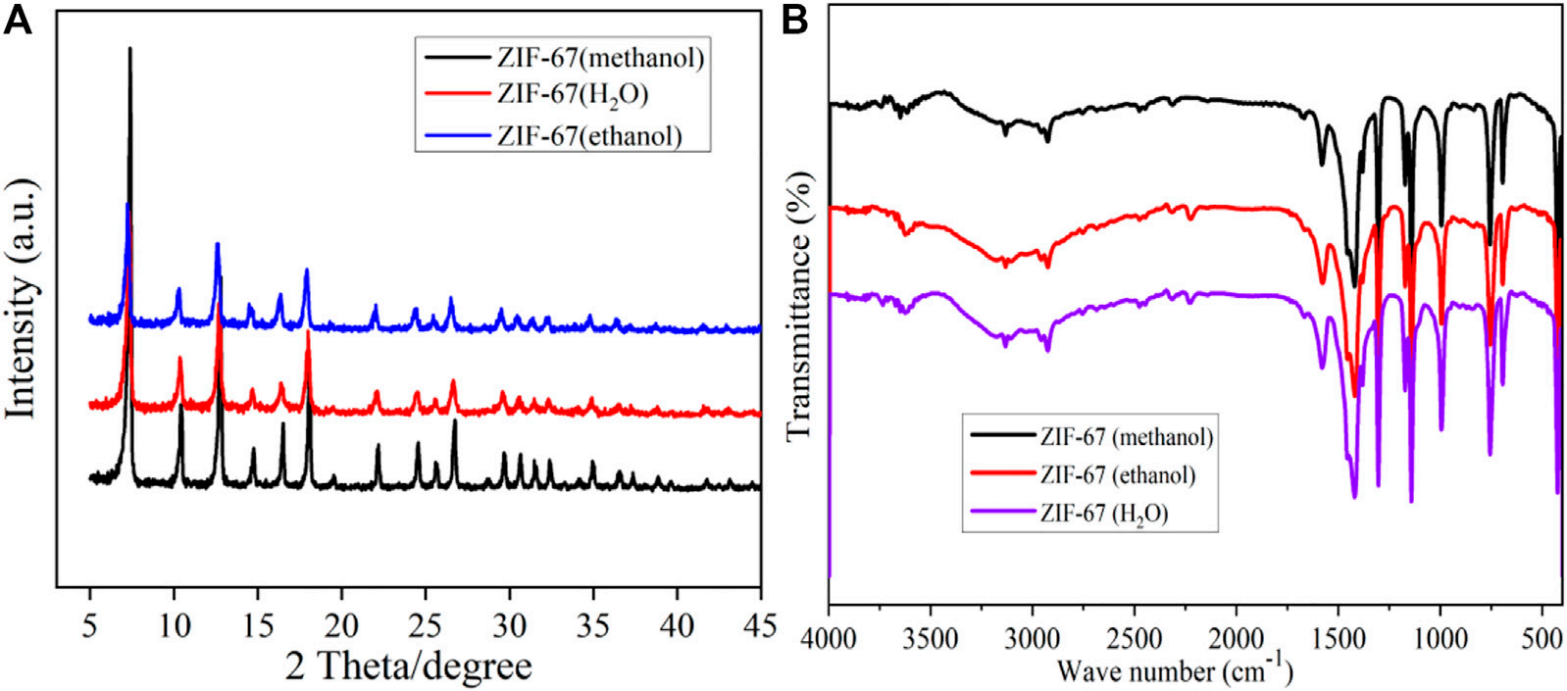
The XRD **(A)** and FT-IR **(B)** of ZIF-67.

It can be seen from Figure 1A that the diffraction peaks of ZIF-67 prepared with three different solvents are corresponding, and there is no apparent difference, indicating that ZIF-67 has been synthesized. However, the diffraction peak of ZIF-67 (methanol) is the highest, suggesting that it has better crystallinity, which is consistent with the SEM and TEM characterization results (Fu et al., 2022).In Figure 1B, the Infrared spectra of ZIF-67 (CH_3_OH), ZIF-67 (water), and ZIF-67 (C_2_H_5_OH) are the same, indicating that ZIF-67 material has been successfully synthesized. The characteristic absorption band in the figure is mainly from the organic ligand 2-methylimidazole. The typical absorption band in the range of 500–1,500 cm^-1^ is also primarily caused by the stretching and bending vibration of the imidazole ring. The stretching mode of the C=N bond in 2-methylimidazole generates the characteristic absorption peak at 1,580 cm^−1^. The distinct absorption peaks at 2,925 cm^-1^ and 3,132 cm^-1^ come from the extension of the C-H bond of the fat chain in the imidazole ring and 2-methylimidazole, respectively (Mahmoodi et al., 2019).

**FIGURE 2:**
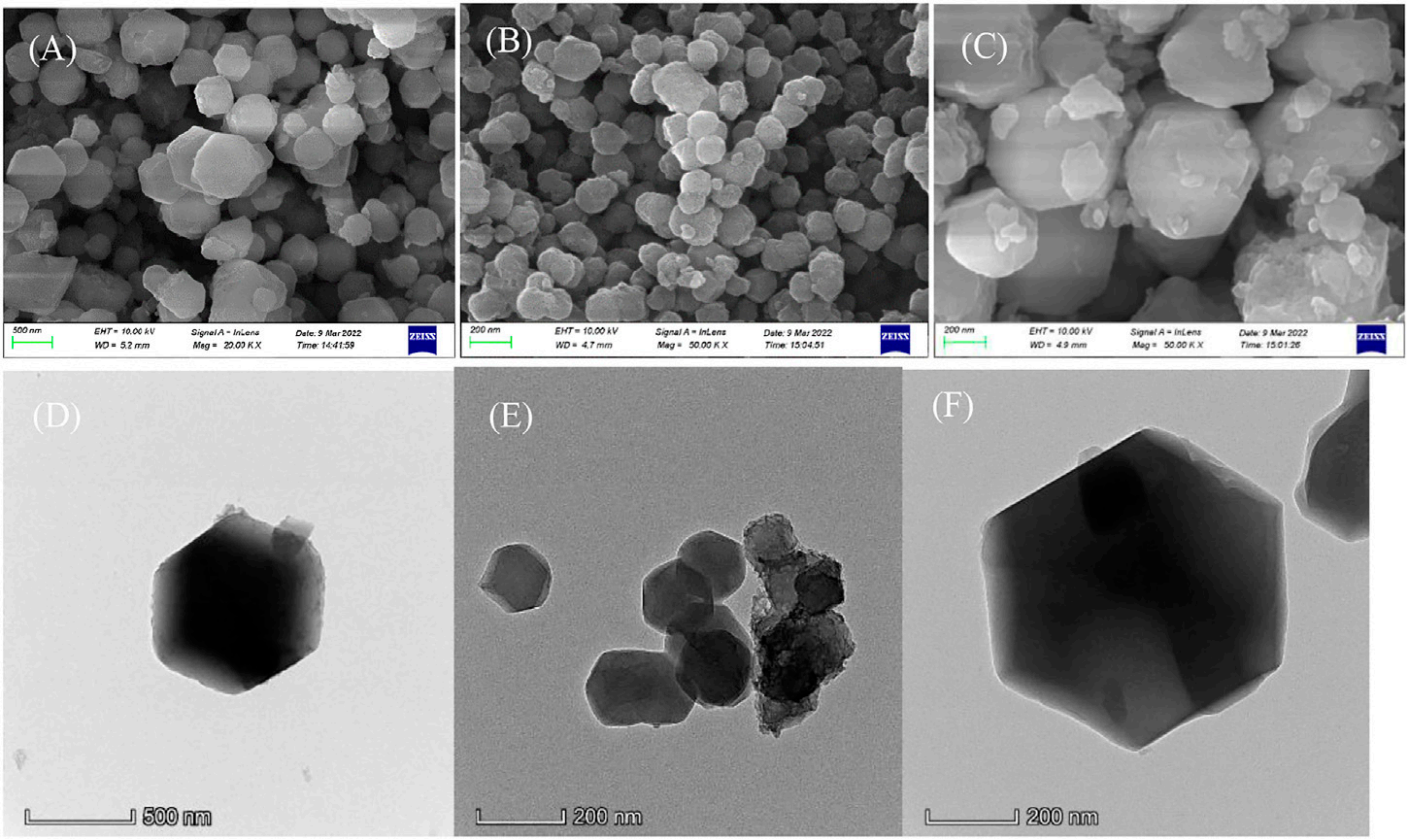
The SEM **(A)** and TEM **(D)** of ZIF-67 (CH_3_OH); SEM **(B)** and TEM **(E)** of ZIF-67 (C_2_H_5_OH); SEM **(C)** and TEM **(F)** of ZIF-67 (H_2_O).

The surface of ZIF-67 (CH_3_OH) is smooth. As shown in Figure 2A, ZIF-67 (CH_3_OH) presents a regular dodecahedral structure with a particle size of about 300–500 nm and no accumulation. However, the surface of ZIF-67 (C_2_H_5_OH) is relatively rough, presenting an irregular spherical shape with a particle size of 100–200 nm and less agglomeration (in Figure 2B). The situation of ZIF-67 (H_2_O) is poorSome particles are broken, indicating that it is inappropriate to use water as a solvent (in Figure 2C).

The TEM diagram in Figure 2D shows that ZIF-67 prepared with methanol as solvent has a perfect crystal form and will not agglomerate. ZIF-67 (C2H5OH) has apparent accumulation, which has an impact on adsorption (Figure 2E). Although the appearance of ZIF-67 (H2O) is regular, it is regrettable that it is broken (Figure 2F).

Table 1 shows the specific surface area, pore volume, and pore diameter data of ZIF-67 prepared with three different solvents. From this, we know that ZIF-67 (CH_3_OH) has the largest specific surface area, the enormous pore volume, and the average pore size, which is more suitable for the adsorption of heavy metals. Therefore, we used methanol as a solvent to synthesize magnetic adsorbent in subsequent experiments.

**TABLE 1:** Textural properties of ZIF-67.

Sample	S_BET_ (m^2^/g)	Pore volume (cm^3^/g)	Pore diameter (nm)
ZIF-67 (CH_3_OH)	1,283	0.6379	1.99
ZIF-67 (C_2_H_5_OH)	918.6	0.5535	2.41
ZIF-67 (H_2_O)	1,055	0.5306	2.01

### 3.2 Characterization of Fe_3_O_4_@ZIF-67

**FIGURE 3:**
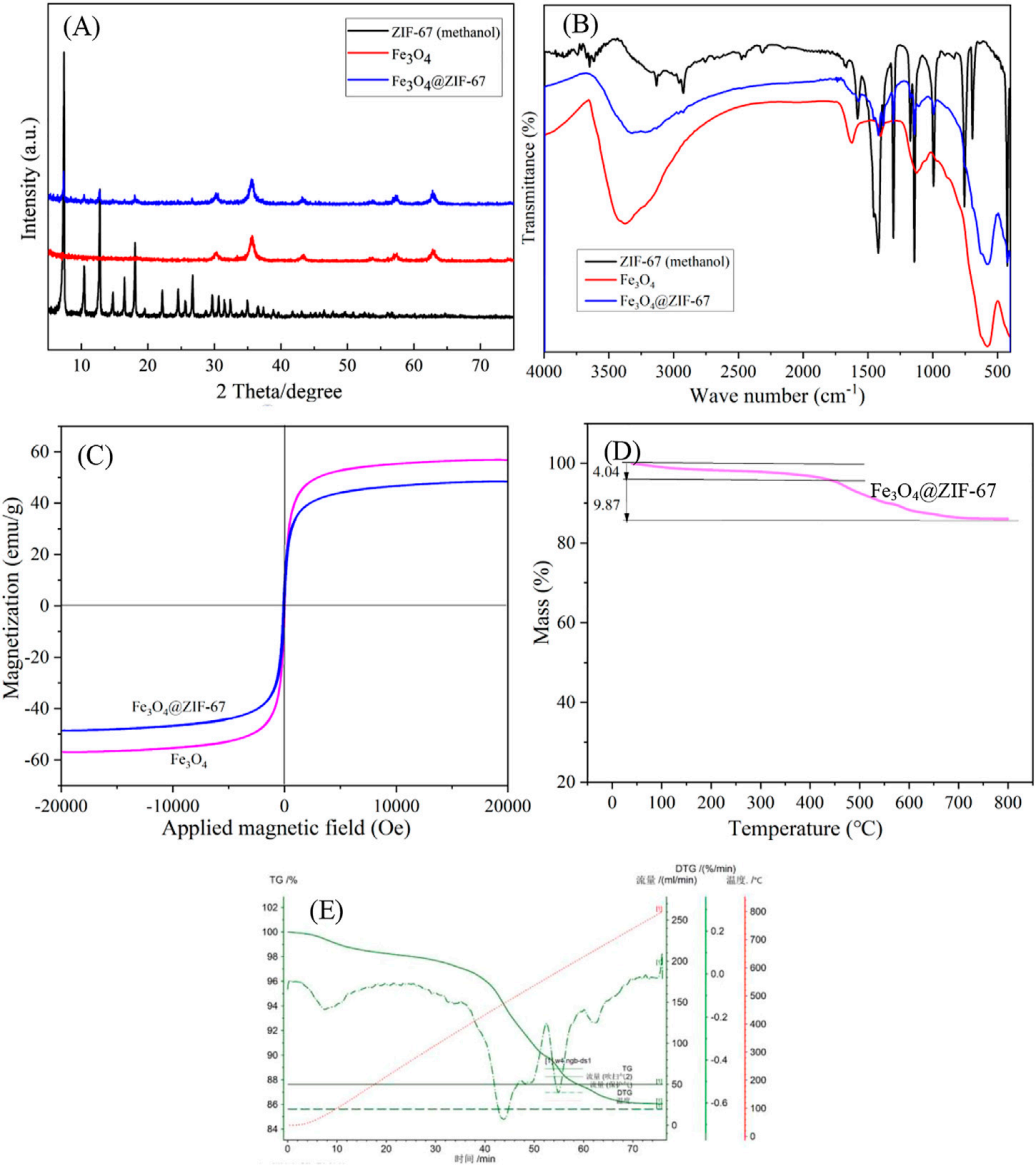
The XRD **(A)**, FT-IR **(B)**, VSM **(C)**, and TGA **(D, E)** of Fe_3_O_4_@ZIF-67.

In the XRD diagram (Figure 3A), the diffraction peaks at 7.5°, 12.8°, and 17.9° belong to the characteristic peak of ZIF-67. The diffraction peaks of 30.2°, 35.6°, 43.2°, 57.3° and 62.8° are the diffraction peaks of Fe_3_O_4_. There are characteristic peaks of ZIF-67 and Fe_3_O_4_ nanoparticles in the diffraction peak of Fe_3_O_4_@ZIF-67, indicating that the preparation of Fe_3_O_4_@ZIF-67 is successful (Sun et al., 2021; Al-Fakih et al., 2022).

According to the FT-IR spectrum (Figure 3B), an apparent characteristic absorption band at 580 cm^-1^ is Fe-O stretching vibration. The absorption bands at 1,620 and 3,369 cm^-1^ are O-H deformation vibration and stretching vibration, respectively, indicating that the surface of Fe_3_O_4_ nanoparticles is rich in hydroxyl (Liu et al., 2019). ZIF-67 (methanol) and Fe_3_O_4_@ZIF-67 The characteristic absorption bands of 2-methylimidazole are mainly from the organic ligand. The distinct absorption peaks in the range of 500–1,500 cm^-1^ are primarily caused by the stretching and bending vibration of the imidazole ring.

It can be seen from the VSM diagram that Fe_3_O_4_@ZIF-67 and Fe_3_O_4_ are ferromagnetic (Figure 3C). And their solid magnetic response to the external magnetic field can be observed. The saturation magnetization of Fe_3_O_4_@ZIF-67 (48 emu/g) is slightly less than Fe_3_O_4_ (56 emu/g), and strong magnetism shows that it can be recovered by magnetic separation.

Thermogravimetric analysis (TGA, Figures 3D, E) characterizes the thermal stability of adsorbent Fe_3_O_4_@ZIF-67. The carrier gas used in the TGA is Ar, and the carrier gas flow rate is 20 mL/min. When the temperature is lower than 440°C, the mass loss of A is only 4.04%. When the temperature reaches 800°C, the mass of Fe_3_O_4_@ZIF-67 is further lost by 9.87%. It shows that the adsorbent has good thermal stability.

**FIGURE 4:**
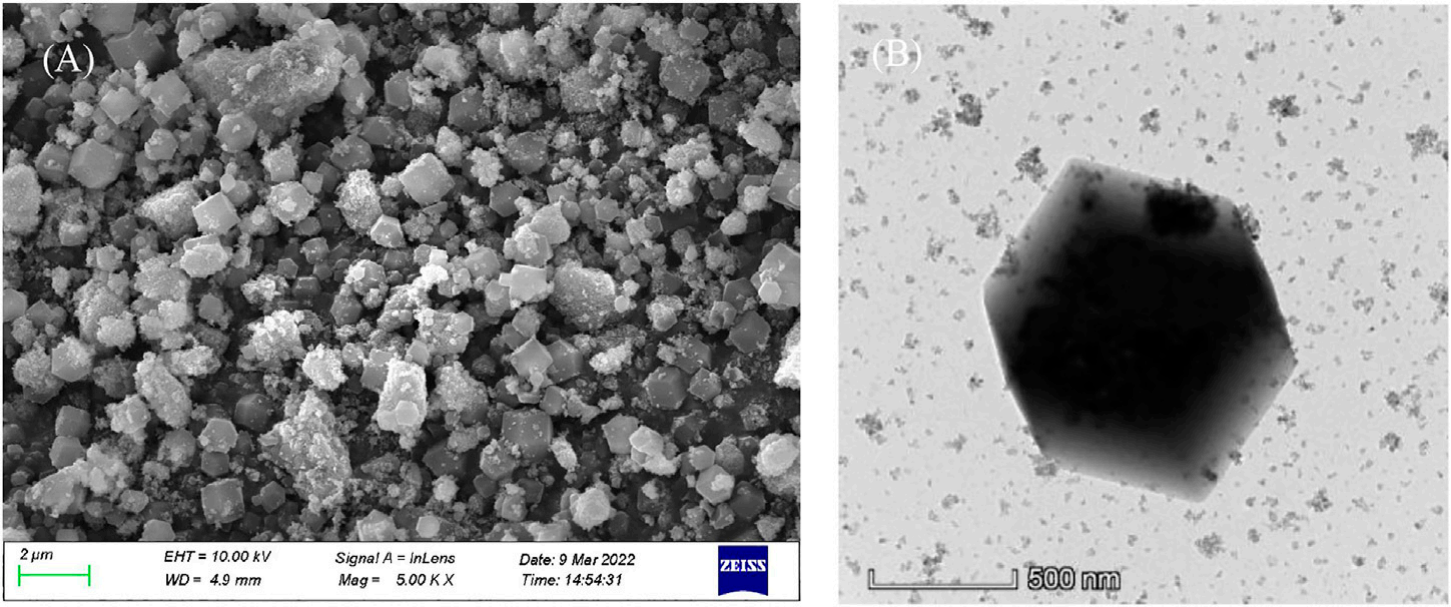
The SEM **(A)** and TEM **(B)** of Fe_3_O_4_@ZIF-67.

As shown in Figure 4A, the SEM legend of Fe_3_O_4_@ZIF-67 shows that it is similar to ZIF-67, with a rough surface and agglomeration. From the TEM diagram (in Figure 4B), we can observe the typical rhombic structure, which indicates that the form of ZIF-67 can be well retained in the Fe_3_O_4_ modification process. It ensures its good magnetism, which is conducive to separation and reuse.

**FIGURE 5:**
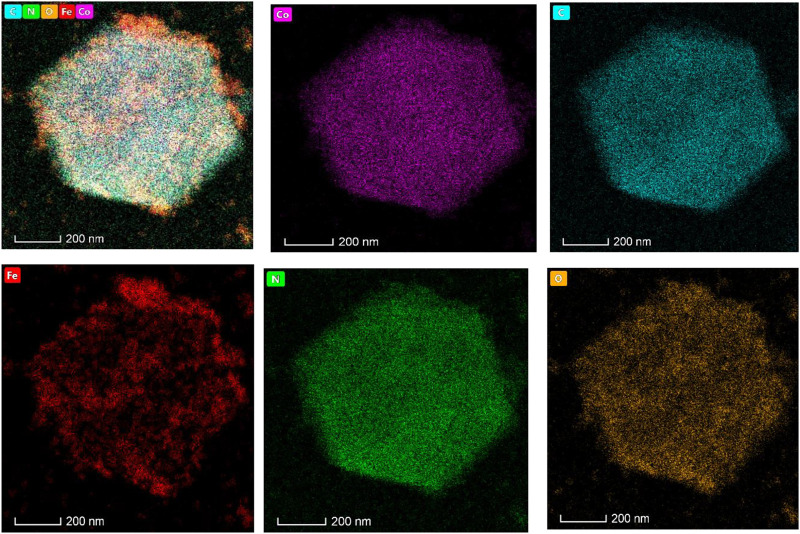
The element distribution mapping of Fe_3_O_4_@ZIF-67.

In the element distribution diagram (Figure 5), Co, C, N, and O elements are uniformly doped in the adsorbent. The Fe element is distributed in the pore channel of the adsorbent, forming a package structure. According to our design, four pieces of Co, N, C, and O form ZIF-67 and are uniformly distributed, while magnetic Fe_3_O_4_ is distributed in the channel of ZIF-67 as the magnetic core. From the element distribution of Fe, we can see that Fe is not entirely covered in the crystal of ZIF-67, proving that the adsorbent preparation is successful.

Table 2 gives the specific surface area, pore volume, and aperture information of ZIF-67 (CH_3_OH), Fe_3_O_4,_ and Fe_3_O_4_@ZIF-67 in more detail. The specific surface area of Fe_3_O_4_@ZIF-67 (230.1 m^2^/g) is much smaller than that of ZIF-67 (CH_3_OH) (1,283 m^2^/g), mainly because Fe_3_O_4_ occupies the pore channel of ZIF-67 (CH_3_OH), resulting in the reduction of the specific surface area of Fe_3_O_4_@ZIF-67. However, the surface area of Fe_3_O_4_@ZIF-67 still reaches 230.1 m^2^/g, which can still meet the demand for the adsorption of Cu^2+^.

**TABLE 2:** Textural properties of Fe_3_O_4_@ZIF-67.

Sample	S_BET_ (m^2^/g)	Pore volume (cm^3^/g)	Pore diameter (nm)
ZIF-67 (CH_3_OH)	1,283	0.6379	1.99
Fe_3_O_4_	109.1	0.2847	10.5
Fe_3_O_4_@ZIF-67	230.1	0.3024	5.26

### 3.3 Adsorption experiments

Based on previous research literature, we preliminarily determined to complete the experiment at room temperature in the adsorption parameters. In addition, the pH value of the initial solution is adjusted to 5 with acetic acid for adsorption because the adsorption effect under this condition is usually the best (Zolgharnein et al., 2022). For this reason, we mainly explored the influence of adsorption time, adsorbent dosage, and initial concentration on the adsorption process.

#### 3.3.1 Effect of time on adsorption

**FIGURE 6:**
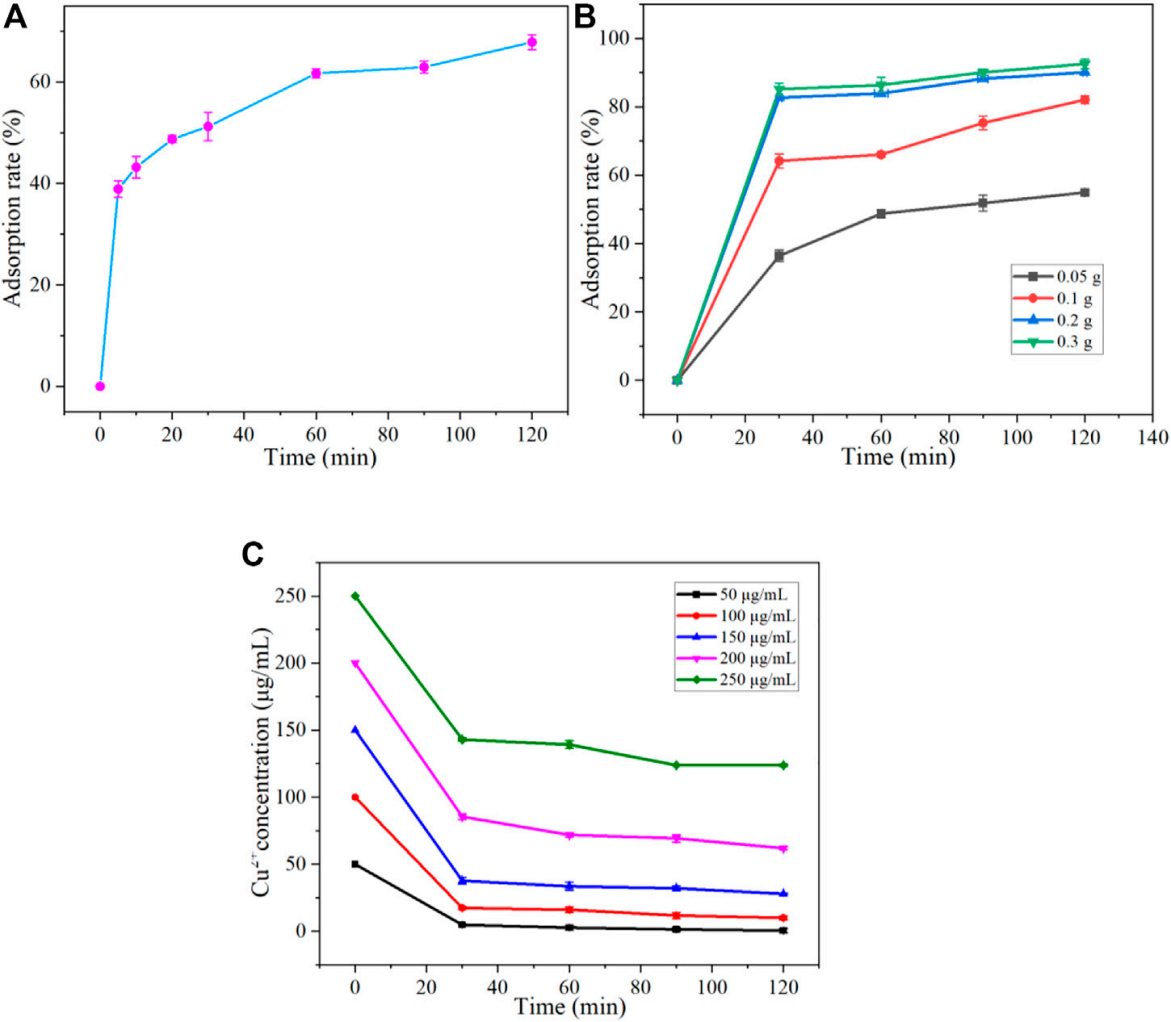
Effect of Adsorption time **(A)**, Adsorption dose **(B)**, and Initial concentration **(C)** on adsorption of Cu^2+^ by Fe_3_O_4_@ZIF-67.

To investigate the effect of adsorption time on the adsorption of Cu^2+^, we set the initial concentration of Cu^2+^ and 0.1 g adsorbent dosage at 25°C for PH = 5. The adsorption rates were measured at 5, 10, 20, 30, 60, 90, and 120 min, respectively. The results are shown in Figure 6A. The adsorption rate increased with adsorption time. The rapid adsorption stage of the adsorbent for Cu^2+^ is 60 min before the initial time. In the range of 0–10 min, the adsorption rate changes the most. However, within 60–120 min, the adsorption rate increased slowly. At the same time, at 120 min, the adsorption rate reached the maximum value of 67.87% in the observation range.

#### 3.3.2 Effect of adsorbent dosage on adsorption

Furthermore, the effect of adsorption dose on the adsorption of Cu^2+^ was investigated. Our initial concentration of Cu^2+^ is about 100 *μ*g/mL at 25°C for PH = 5, and the added adsorption dose is 0.05, 0.1, 0.2, and 0.3 g, respectively. Monitor the adsorption rate of the adsorbent at 30, 60, 90, and 120 min, and the results are shown in Figure 6B. The adsorption rate increases with the increase of adsorption dose until it reaches a higher adsorption rate and remains stable. At 0–30 min, the adsorption rate increased rapidly, which showed that the adsorbent rapidly adsorbed Cu2+ in the first 30 min. At 120 min, the adsorption rate also reached 54.92%, 82.05%, 90.06%, and 92.53% when the adsorption dose was 0.05 g, 0.1 g, 0.2 g, and 0.3 g, respectively. With adsorption doses of 0.2 g and 0.3 g, although the adsorption rate will not increase significantly, it will grow slowly, which indicates that when the amount of adsorbent is large, Fe_3_O_4_@ZIF-67 The adsorption rate can reach more than 90%. If the experimental conditions and error factors can be controlled well, the adsorption rate can get 100%, matching the adsorption effect of completely adsorbing Cu^2+^.

#### 3.3.3 Effect of Cu^2+^ concentration on adsorption

When the amount of Fe_3_O_4_@ZIF-67 is 0.2 g at 25°C for PH = 5, the initial concentration of Cu^2+^ solution is 50, 100, 150, 200, and 250 *μ*g/mL, respectively. Take 50 mL of solutions with different initial attention, add 0.2 g of Fe_3_O_4_@ZIF-67, put it into a water bath constant temperature shaker, and shake it. The effect of the initial concentration of Cu^2+^ solution on the adsorption rate was investigated at 30, 60, 90, and 120 min, respectively. The results are shown in Figure 6C. During the first 30 min, the concentration of Cu^2+^ solution dropped sharply, indicating that A was rapidly adsorbing Cu^2+^. In comparison, from 30 min to 120 min, the concentration of Cu^2+^ solution decreased slowly. When the concentration of Cu^2+^ solution is 250 *μ*g/mL, the adsorption equilibrium has been reached after 90 min, so the image is close to a straight line. At 120 min, the adsorption rate of Fe_3_O_4_@ZIF-67 on the five solutions with different initial concentrations of Cu^2+^ also reached the maximum value in the observation adsorption time. Fe_3_O_4_@ZIF-67 initial concentrations of 50, 100, 150, 200, and 250 *μ*g/mL in Cu^2+^ solution, the adsorption rate reaches 99.04%, 81.39%, 69.03%, 65.32%, and 50.49%, respectively. Therefore, it can be concluded that when the adsorption dose of F was 0.2 g, the adsorption rate decreased with the increase of the initial concentration of the solution. Within the observation range, when the initial concentration of the solution is 50 *μ*g/mL, the adsorption rate can reach 93.4% in 30 min, and the adsorption effect is pronounced. It indicates that the adsorbent has a good adsorption and removal effect on a low concentration of Cu^2+^.

### 3.4 Reuse of adsorbent

**FIGURE 7:**
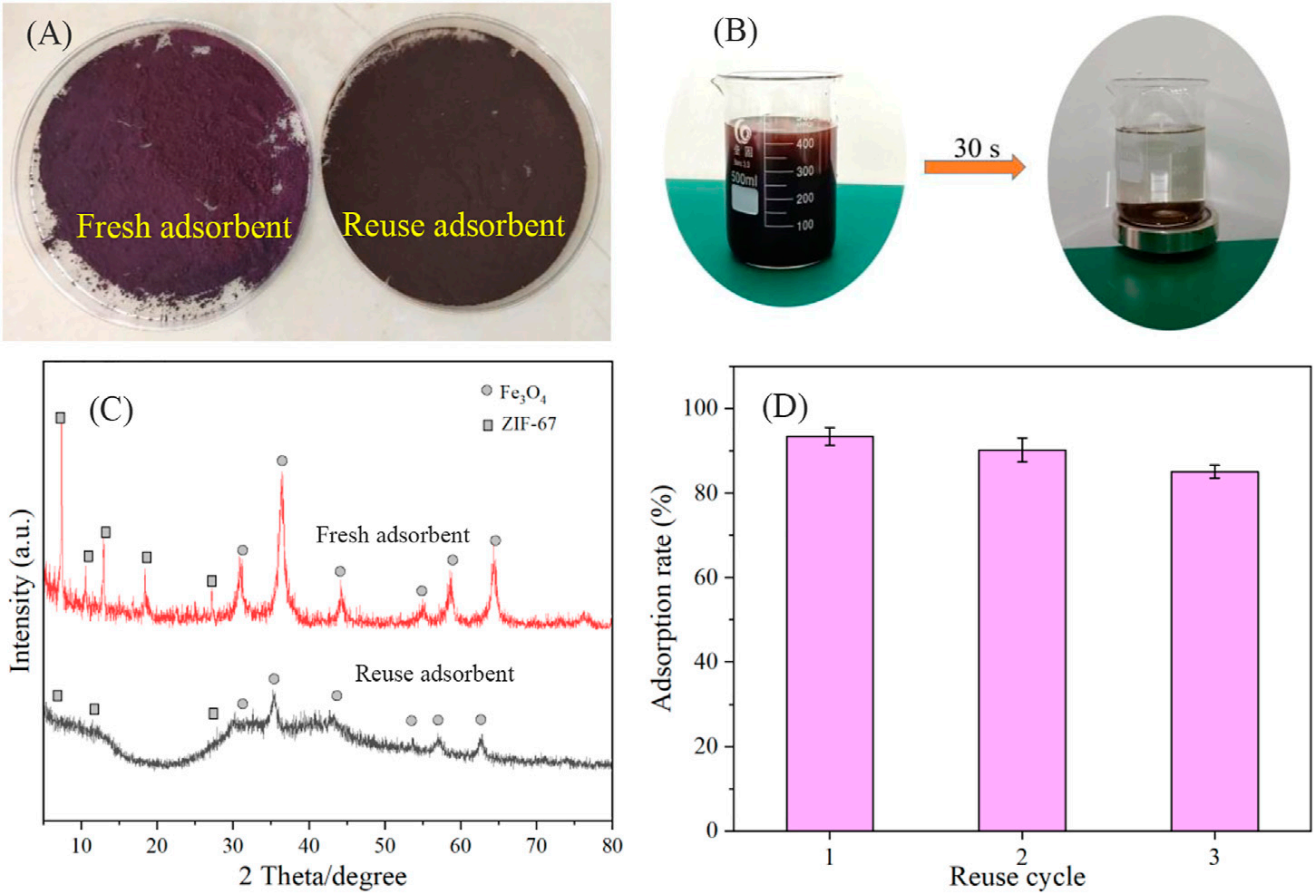
The color contrast of adsorbent before and after adsorption **(A)**, magnetic property of adsorbent **(B)**, XRD contrast of adsorbent before and after adsorption **(C)**, and reusability of Fe_3_O_4_@ZIF-67 **(D)**.

After using the adsorbent for three cycles, we compared the recovered adsorbent with the fresh catalyst. First, in Figure 7A, we can see that the new adsorbent appears purplish red, while the used adsorbent appears black, indicating that some changes may have occurred in the used adsorbent. Secondly, a magnetic recovery experiment was carried out on the used adsorbent. The results show that the adsorbent can be quickly recovered through the magnet in only 30 seconds (Figure 7B). Finally, we characterized the catalyst before and after use with XRD. Although the adsorbent after use has the original adsorbent’s characteristic peak, the diffraction peak’s abundance is significantly reduced, indicating that the structure may have changed (Figure 7C). This is consistent with the result that the adsorbent only maintained three cycles (Figure 7D).

### 3.5 Adsorption conditions

As seen from Table 3, Fe_3_O_4_@ZIF-67 has excellent magnetic properties (48.0 emu/g), which is beneficial to the rapid recovery and utilization of adsorbent. At the same time, in a short time (0.5 h), the adsorption equilibrium can be reached quickly, and the maximum adsorption capacity is 55.2 mg/g, which meets the demand of rapid adsorption to measure Cu^2+^.

**TABLE 3:** Comparison of adsorption conditions of different adsorbents.

Sample	Magnetic hysteresis loop (emu/g)	T (°C)	PH	Time (h)	Adsorption capacity (mg/g)	Ref
GO/Fe_3_O_4_	31.0	20	5.3	24	18.26	Li et al. (2012)
CTPP beads	—	27	4.5	1.67	26.06	Ngah and Fatinathan (2010)
NMCMs	30.1	25	5.0	20	65.8	Peng et al. (2014)
TMCS	17.6	15	6.0	8.0	66.7	Zhou et al. (2009)
Fe_3_O_4_@ZIF-67	48.0	25	5.0	0.5	55.2	This study

## 4 Conclusion

In this work, Fe_3_O_4_@ZIF-67 was successfully synthesized by magnetic Fe_3_O_4_ encapsulated in MOF. According to the physical and chemical properties, Fe_3_O_4_@ZIF-67 has a porous structure with a surface area of 230.1 m^2^/g and has good magnetic properties and thermal stability. Through the experimental study of Cu^2+^ adsorption, the MOF-modified material can effectively adsorb at pH 5.0. The adsorption rate of low-concentration Cu^2+^ wastewater (50 *μ*g/mL) reached 93.4% within 30 min at 25°C. At the same time, the saturation magnetization of Fe_3_O_4_@ZIF-67 is up to 48.0 emu/g, which the external magnetic field can quickly recover within 30 s. It shows that A is expected to play a more important role in heavy metal adsorption and has more essential application prospects.

## Data Availability

The original contributions presented in the study are included in the article/supplementary material, further inquiries can be directed to the corresponding authors.
